# Localized cortical chronic traumatic encephalopathy pathology after single, severe axonal injury in human brain

**DOI:** 10.1007/s00401-016-1649-7

**Published:** 2016-11-24

**Authors:** Sharon B. Shively, Sarah L. Edgerton, Diego Iacono, Dushyant P. Purohit, Bao-Xi Qu, Vahram Haroutunian, Kenneth L. Davis, Ramon Diaz-Arrastia, Daniel P. Perl

**Affiliations:** 1 Department of Pathology, F. Edward Hébert School of Medicine, Uniformed Services University of the Health Sciences, 4301 Jones Bridge Road, Bethesda, MD 20814 USA; 2 Center for Neuroscience and Regenerative Medicine, 4301 Jones Bridge Road, Bethesda, MD 20814 USA; 3 Henry M. Jackson Foundation for the Advancement of Military Medicine, 6720 Rockledge Drive, Bethesda, MD 20817 USA; 4 Department of Pathology, Icahn School of Medicine at Mount Sinai, 1468 Madison Avenue, New York, NY 10029 USA; 5 Department of Neurology, F. Edward Hébert School of Medicine, Uniformed Services University of the Health Sciences, 4301 Jones Bridge Road, Bethesda, MD 20814 USA; 6 Department of Psychiatry, Icahn School of Medicine at Mount Sinai, 1 Gustave L. Levy Place, New York, NY 10029 USA; 7 Department of Neuroscience, Icahn School of Medicine at Mount Sinai, 1 Gustave L. Levy Place, New York, NY 10029 USA; 8 Mental Illness Research Education and Clinical Center, James J. Peters Veterans Affairs Medical Center, 130 West Kingsbridge Road, Bronx, NY 10468 USA; 9 Department of Pharmacological Sciences, Icahn School of Medicine at Mount Sinai, 1 Gustave L. Levy Place, New York, NY 10029 USA

**Keywords:** Traumatic brain injury, Chronic traumatic encephalopathy, Axonal injury, Tau, Neurofibrillary tangle, β-Amyloid plaque

## Abstract

Chronic traumatic encephalopathy (CTE) is a neurodegenerative disease associated with repetitive mild impact traumatic brain injury from contact sports. Recently, a consensus panel defined the pathognomonic lesion for CTE as accumulations of abnormally hyperphosphorylated tau (p-tau) in neurons (neurofibrillary tangles), astrocytes and cell processes distributed around small blood vessels at sulcal depths in irregular patterns within the cortex. The pathophysiological mechanism for this lesion is unknown. Moreover, a subset of CTE cases harbors cortical β-amyloid plaques. In this study, we analyzed postmortem brain tissues from five institutionalized patients with schizophrenia and history of surgical leucotomy with subsequent survival of at least another 40 years. Because leucotomy involves severing axons bilaterally in prefrontal cortex, this surgical procedure represents a human model of single traumatic brain injury with severe axonal damage and no external impact. We examined cortical tissues at the leucotomy site and at both prefrontal cortex rostral and frontal cortex caudal to the leucotomy site. For comparison, we analyzed brain tissues at equivalent neuroanatomical sites from non-leucotomized patients with schizophrenia, matched in age and gender. All five leucotomy cases revealed severe white matter damage with dense astrogliosis at the axotomy site and also neurofibrillary tangles and p-tau immunoreactive neurites in the overlying gray matter. Four cases displayed p-tau immunoreactivity in neurons, astrocytes and cell processes encompassing blood vessels at cortical sulcal depths in irregular patterns, similar to CTE. The three cases with apolipoprotein E ε4 haplotype showed scattered β-amyloid plaques in the overlying gray matter, but not the two cases with apolipoprotein E ε3/3 genotype. Brain tissue samples from prefrontal cortex rostral and frontal cortex caudal to the leucotomy site, and all cortical samples from the non-leucotomized patients, showed minimal p-tau and β-amyloid pathology. These findings suggest that chronic axonal damage contributes to the unique pathology of CTE over time.

## Introduction

In 2010 an estimated 2.5 million persons suffered from traumatic brain injury (TBI) in the United States, of whom 50,000 died from the injury and another 2,80,000 required hospitalization [36]. For survivors, TBI is a known environmental risk factor for the development of dementia later in life. Multiple studies provide evidence that single moderate or severe TBI increases the likelihood of eventual presentation with clinical signs and symptoms of neurodegeneration, such as Alzheimer Disease (AD) [7, 29, 35]. Such studies typically did not include postmortem examinations of brain tissues. Rather, the medical literature scantily addressed chronic neuropathological sequelae after TBI. One recent autopsy study of 39 patients with long-term survival (1–47 years) after single moderate or severe TBI, however, showed widespread β-amyloid plaques and neurofibrillary tangles (NFTs) in the brains of approximately one-third of patients [17]. Whether single mild TBI (concussion) similarly confers increased risk of developing dementia with neuropathological abnormalities in susceptible individuals remains undetermined.

For contact sport athletes with repetitive mild TBIs or subconcussive events, such as boxers and American football players, studies of postmortem brains from symptomatic patients revealed a progressive neurodegenerative disorder with distinctive neuropathological features, called chronic traumatic encephalopathy (CTE) [4, 27]. Often presenting with symptoms years after cessation of contact sport participation, CTE patients tend to exhibit initially behavioral and mood abnormalities, cognitive impairments and chronic headache, whereas in advanced stages, these patients deteriorate to frank dementia [28, 33, 45]. Like other neurodegenerative tauopathies, disease progression relates to the burgeoning spread of abnormally hyperphosphorylated tau protein (p-tau) [28]. In the first consensus meeting to define neuropathological criteria for CTE, the participants established the pathognomonic lesion as p-tau accumulation in neurons (NFTs), astrocytes and cell processes, with predilection around small blood vessels, at sulcal depths in irregular cortical spatial patterns [26]. The biological mechanism underlying this observation is unknown. In addition, a subset of reported CTE cases demonstrated diffuse, or more rarely neuritic, β-amyloid plaques [4, 26, 39, 44].

TBIs commonly involve axonal injury, a prominent factor contributing to symptomatology and one of multiple possible lesions in the human brain consequent to head impact [15, 34, 46]. Therefore, we identified a human model that isolates the axonal damage component of TBI, that is, surgical leucotomy, and then tested the hypothesis that chronic axonal injury can result in neurodegenerative changes, such as NFTs and β-amyloid plaques. We analyzed postmortem brain tissues from five institutionalized patients with schizophrenia, who had undergone bilateral prefrontal leucotomy as therapy prior to 1953 and then lived at least another 40 years (Table 1). Since leucotomy involves severing axons in the prefrontal cortex, this surgical procedure represents a single iatrogenic TBI with severe axonal damage and no external cortical impact. We examined cortical tissues at the leucotomy site, prefrontal cortex rostral and frontal cortex caudal to the leucotomy site, and hippocampus for evidence of p-tau and β-amyloid accumulations. We also examined postmortem cortical brain tissues of five patients with schizophrenia from the same institution, who had not undergone leucotomies in their lifetimes, matched in gender and approximate age (±4 years) to the leucotomy patients (Table 1). Moreover, we determined the apolipoprotein E (*APOE*) genotype for all ten patients. We found p-tau accumulations in the gray matter at the axotomy site in all five leucotomy patients, with four cases showing the pathognomonic p-tau pattern of CTE. Scattered β-amyloid plaques also occupied the gray matter at the lesion site, but in the three leucotomy patients with *APOE* ε4 haplotype. We observed minimal p-tau and β-amyloid pathology in the cortical samples rostral and caudal to the leucotomy site and in all cortical samples from the non-leucotomized schizophrenia patients.

**Table 1:** Demographic features of five schizophrenia patients in leucotomy cohort and five schizophrenia patients without leucotomy in comparative cohort

Case number (leucotomy)	Age (years)	Gender	Case number (no leucotomy)	Age (years)	Gender
1	67	Male	6	67	Male
2	70	Female	7	70	Female
3	77	Female	8	77	Female
4	87	Male	9	83	Male
5	89	Female	10	86	Female

## Materials and methods

All brain tissues for this study were obtained from the brain bank at the Pilgrim Psychiatric Center in West Brentwood, NY. The Pilgrim Psychiatric Center opened in 1931 as a site for care and treatment of patients with chronic psychiatric conditions. The patient population continually expanded such that the institution cared for almost 14, 000 patients in 1954. Many resident patients suffered from chronic schizophrenia. In the late 1940s until 1953, surgeons commonly performed bilateral prefrontal leucotomy as therapy for intractable symptoms. Currently available clinical records do not detail the surgical procedure, but based on autopsy findings and medical literature, the surgeons most likely used bilateral burr holes at the coronal suture between the frontal and parietal bones to access the brain [5]. In recent years, many patients have been discharged to the care of local community mental health centers. Several hundred patients, however, were too impaired for community living and thus remained as inpatients. Drs. Kenneth Davis and Vahram Haroutunian established the brain bank for the Pilgrim Psychiatric Center, with Dr. Daniel Perl serving as neuropathologist.

We sampled brain specimens fixed in formalin, embedded these tissues in paraffin and then sectioned the tissue blocks at 5 μm thickness. For the leucotomy postmortem brain tissues, the samples comprised cortical tissues cut in the coronal plane at the leucotomy site, prefrontal cortex rostral and frontal cortex caudal to the leucotomy site, and hippocampus; for the non-leucotomized postmortem brain tissues, the samples included equivalent cortical neuroanatomical sites. We conducted hematoxylin and eosin (H&E) stains on tissue sections for general morphology/structure and immunohistochemistry with antibodies detecting glial fibrillary acidic protein (GFAP, astrocyte marker, mouse anti-human monoclonal antibody GA5, Leica Biosystems, PA0026), abnormally hyperphosphorylated tau (AT8, pS199/S202/T205; mouse anti-human monoclonal antibody, Thermo Scientific, MN1020), β-amyloid (mouse anti-human monoclonal antibody, clone 6F/3D, Leica Biosystems, NCL-B-Amyloid) and antigen CD68 (marker of macrophages and activated microglia, mouse anti-human monoclonal antibody clone 514H12, Leica Biosystems, PA0273). Immunohistochemistry for these antibodies was performed on a Leica Bond III automated immunostainer with a diaminobenzidine (DAB) chromogen detection system (Leica Biosystems, DS9800, Buffalo Grove, IL).

We also performed immunohistochemistry experiments with two other antibodies against abnormally hyperphosphorylated tau (CP13, pS202; PHF1, pS396/S404). Briefly, tissue sections were first deparaffinized and rehydrated. For antigen retrieval, tissue sections were immersed in sodium citrate buffer (pH 6) at boiling temperature for 6 min and then cooled to room temperature. To quench endogenous peroxidase activity, tissue sections were placed in a solution of 0.5% H_2_O_2_ in methanol for 1 h. Tissue sections were incubated in blocking solution, comprised of 5% bovine serum albumin in phosphate-buffered saline (pH 7.4), for 1 h at room temperature. The primary antibodies were diluted in block solution (CP13, mouse anti-human monoclonal antibody, 1:1000; PHF1, mouse anti-human monoclonal antibody, 1:1000; gifts from Dr. Peter Davies, Feinstein Institute for Medical Research, Manhasset, NY). The tissue sections were incubated with primary antibody solution in a humidified chamber at 4 °C overnight. Tissue sections were then incubated with secondary antibody, followed by avidin–biotin-complex incubation (Vectastain Elite ABC Kit, mouse IgG, Vector Laboratories, PK-6102) and visualization with DAB (ImmPACT DAB, Vector Laboratories, SK-4105, Burlingame, CA), according to manufacturer instructions. The sections were counterstained with hematoxylin, dehydrated and cleared with xylene before application of mounting media and coverslip.

Tissue samples also underwent modified Bielschowsky silver staining for detection of NFTs and neuritic plaques [8]. We extracted DNA from formalin-fixed brain tissues, according to manufacturer’s instructions (DNeasy Blood & Tissue Kit, QIAGEN, 69504, Valencia, CA). *APOE* genotyping was performed with real-time polymerase chain reaction (RT-PCR) [2].

Two neuropathologists (DI, DPP) conducted blinded evaluations of the stained and immunostained brain tissue samples. This protocol received Institutional Review Board approval prior to initiation of study.

## Results

### Schizophrenia patients with leucotomy

**Fig. 1:**
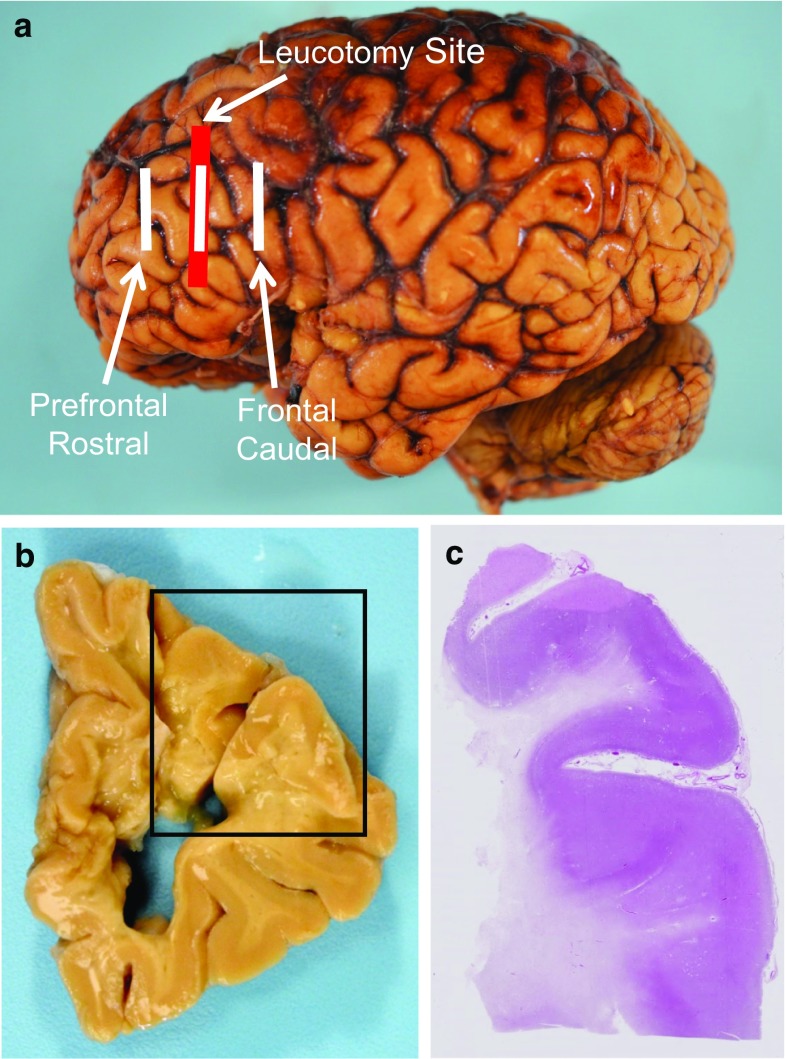
Neuroanatomical sampling sites and macroscopic findings in postmortem brains of leucotomized schizophrenia patients. The *red bar* indicates the approximate location of cortex overlying the leucotomy site; the *white bars* show the three neuroanatomical sites for the cortical/subcortical samples (prefrontal cortex rostral to leucotomy, prefrontal cortex at leucotomy site and frontal cortex caudal to leucotomy) (**a**). Coronal gross appearance at the level of the leucotomy site revealing severe white matter damage, with tissue loss and cyst formation, and relative sparing of gray matter (**b**). H&E stain shows white matter rarefaction and tissue loss, with intact cerebral cortex (**c**). Case 5 (**b**, **c**)

The leucotomized schizophrenia cohort comprised five patients with advanced years at the time of death (mean age 78 years, range 67–89 years, 3 females and 2 males, Table 1). In the standard leucotomy operation, surgeons sectioned axons in white matter of both frontal lobes in the plane of the coronal suture (Fig. 1a) [5]. In all five cases, the leucotomy site in the prefrontal cortex displayed severe white matter damage on both gross examination and H&E staining (Fig. 1b, c). GFAP immunoreactivity indicated replacement of subcortical white matter with dense astrogliosis (Fig. 4a, c, e). The prefrontal cortical sample rostral to the leucotomy site showed intact white matter in case 1. For case 2, GFAP immunohistochemistry showed scattered, sparse astrogliosis. For case 3, GFAP immunoreactivity revealed minimal astrogliosis, and cases 4 and 5 showed moderate astrogliosis. In the frontal cortical samples caudal to the leucotomy site, GFAP immunoreactivity displayed nominal white matter astrogliosis (Table 2). CD68 immunoreactivity was unremarkable in all samples.

**Table 2:** Pathology findings in prefrontal rostral, leucotomy and frontal caudal cortex tissues with subcortical white matter in leucotomized schizophrenia patients and neuroanatomical equivalent sites in non-leucotomized schizophrenia patients

Case number	Prefrontal cortex rostral to leucotomy site	Leucotomy site	Frontal cortex caudal to leucotomy site
Leucotomy			
1	Minimal leptomeningeal CAA	Severe white matter astrogliosis, NFTs, p-tau neurites, β-amyloid plaques	One blood vessel with CAA in gray matter
2	Sparse white matter astrogliosis	Severe white matter astrogliosis, NFTs, p-tau neurites, sulcal depths (astrocytic tangles, p-tau cell processes)	**–**
3	Minimal white matter astrogliosis, scant NFTs and p-tau neurites, one focus subpial p-tau at sulcal depth	Severe white matter astrogliosis, NFTs, p-tau neurites, sulcal depths (astrocytic tangles, p-tau cell processes), β-amyloid plaques	Scant NFTs and p-tau neurites
4	Moderate white matter astrogliosis, scant NFTs and p-tau neurites	Severe white matter astrogliosis, NFTs, p-tau neurites, sulcal depths (astrocytic tangles, p-tau cell processes), β-amyloid plaques	Few β-amyloid plaques
5	Moderate white matter astrogliosis	Severe white matter astrogliosis, NFTs, p-tau neurites, sulcal depths (astrocytic tangles, p-tau cell processes), one focus β-amyloid plaques at sulcal depth	Few β-amyloid plaques
No leucotomy			
6	Few β-amyloid plaques	Scant β-amyloid plaques	*−*
7	*−*	*−*	*−*
8	Scant NFTs	*−*	*−*
9	*−*	*−*	*−*
10	Minimal leptomeningeal CAA	Scant NFTs	*−*

*CAA* Cerebral amyloid angiopathy, *NFTs* neurofibrillary tangles, *p*-*tau* abnormally hyperphosphorylated tau, *−* negative findings

**Fig. 2:**
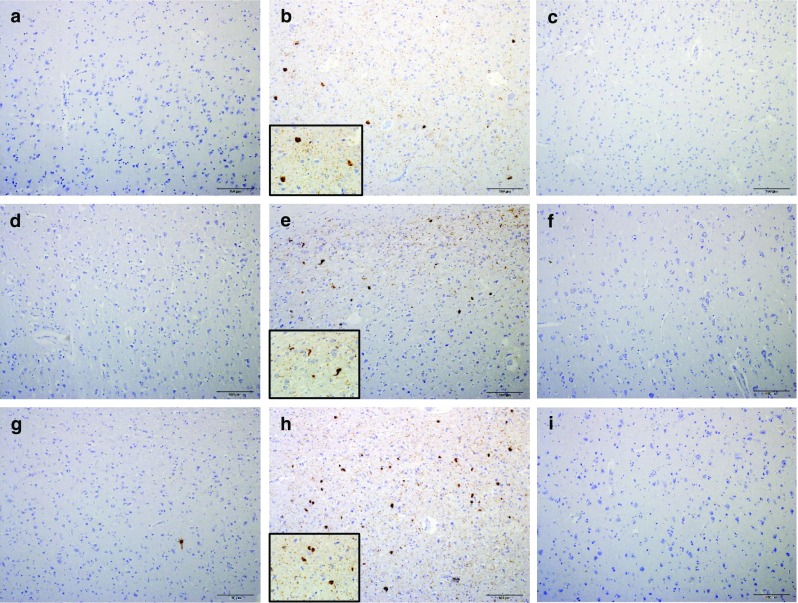
P-tau pathology in cortex adjacent to leucotomy site. NFTs and p-tau immunoreactive neurites in cortex at leucotomy site (**b**, **e**, **h**), but minimal p-tau pathology in cortex rostral (**a**, **d**, **g**) or caudal (**c**, **f**, **i**) to the leucotomy site. Case 1 (**a**, **b**, **c**). Case 2 (**d**–**f**). Case 4 (**g**, **h**, **i**). AT8 immunohistochemistry (**a**–**i**). *Scale bars* **a**–**i** 100 μm

**Fig. 3:**
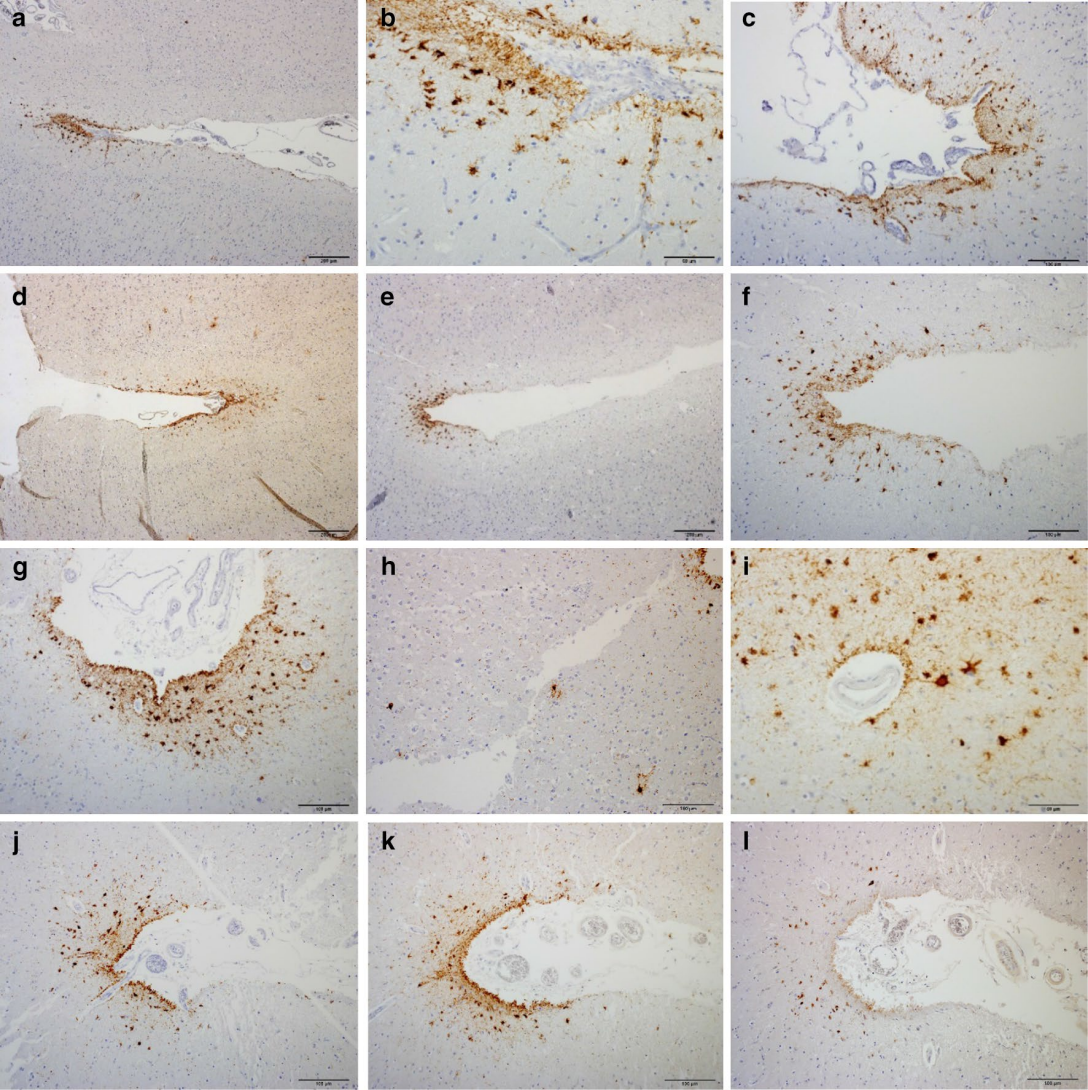
P-tau pathology at sulcal depths in cortex adjacent to leucotomy site. Primarily astrocytic tangles and p-tau immunoreactive cell processes at cortical sulcal depths, including distribution around small blood vessels (**a**–**l**). NFTs at cortical sulcal depth (**h**). AT8, CP13 and PHF1 immunoreactivities at cortical sulcal depth in serial tissue sections (**j**–**l**). Case 3 (**a**–**d**). Case 4 (**e**, **f**). Case 5 (**g**–**l**). AT8 (**a**–**c**, **e**–**h**, **j**), CP13 (**d**, **i**, **k**) and PHF1 (**l**) immunohistochemistry. *Scale bars* **a**, **d**, **e** 200 μm, **c**, **f**–**h**, **j**–**l** 100 μm, **b**, **i** 50 μm

Because the consensus panel for CTE diagnosis recommended AT8 or equivalent p-tau antibodies (CP13 and PHF1), we conducted immunohistochemistry experiments with these three antibodies [26]. All five leucotomy cases showed heightened p-tau immunoreactivity in the cortex at the coronal plane of the axotomy site, specifically NFTs and p-tau immunoreactive neurites (Fig. 2b, e, h). The white matter of cases 1, 4 and 5 revealed rare, isolated p-tau immunoreactive threads. Unlike the cortex at the leucotomy site, the prefrontal rostral cortical tissues of cases 1, 2 and 5 showed no immunoreactivity with AT8, CP13 or PHF1 antibodies, and cases 3 and 4 displayed scant NFTs and p-tau immunoreactive neurites (Fig. 2a, d, g). Frontal cortical tissues caudal to the leucotomy site similarly revealed minimal p-tau pathology. For cases 1, 2, 4 and 5, no p-tau immunoreactivity was detected with AT8, CP13 or PHF1 immunohistochemistry at these sites (Fig. 2c, f, i). AT8 and CP13 immunohistochemistry revealed scant NFTs and p-tau immunoreactive neurites in case 3 (Table 2). For all cases, the AT8 and CP13 antibodies showed correspondent immunoreactivity, whereas PHF1 immunoreactivity revealed less p-tau pathology in these tissue samples (Fig. 3j–l).

**Fig. 4:**
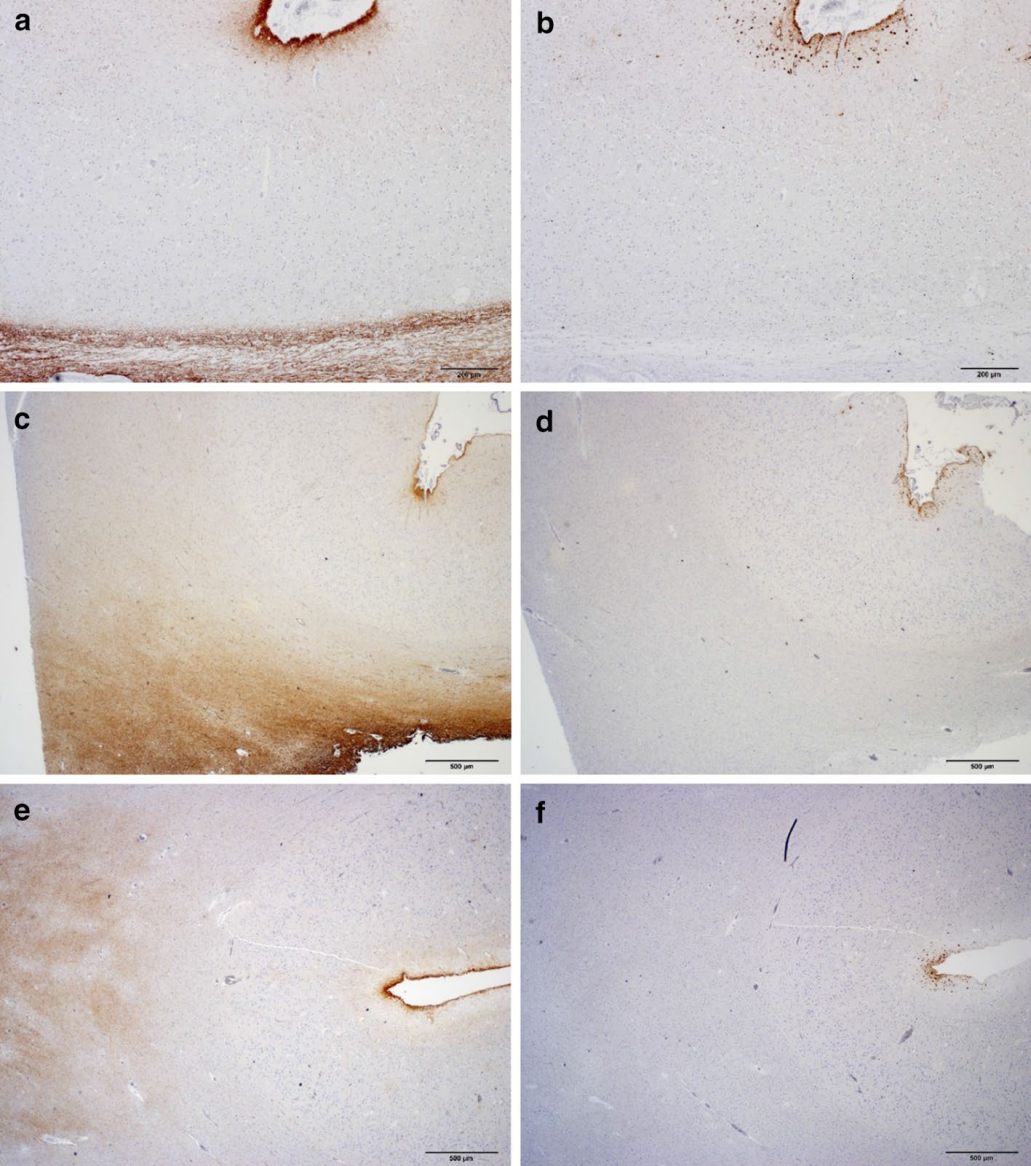
P-tau and GFAP pathology in cortex and subcortical white matter at leucotomy site. GFAP immunoreactivity in white matter showing severe astrogliosis (**a**, **c**, **e**). GFAP and p-tau immunoreactivities at corresponding sulcal depths in serial tissue sections (**a**–**f**). Case 5 (**a**, **b**). Case 3 (**c**, **d**). Case 4 (**e**, **f**). GFAP (**a**, **c**, **e**) and p-tau (**b**, **d**, **f**) immunohistochemistry. *Scale bars* **a**, **b** 200 μm, **c**–**f** 500 μm

In the prefrontal cortex at the leucotomy site, immunohistochemistry with antibodies AT8 and CP13 revealed at least one sulcal depth with primarily astrocytic tangles and p-tau immunoreactive cell processes, including not only perivascular ensheathment, in gray matter of cases 2–5, but also few scattered NFTs either within the cortical layers of the sulcal depth or the two apposing gyri forming the sulcus (Fig. 3a–l). Cortical sulcal depths with p-tau immunoreactivity oriented in irregular patterns, often with one immunopositive sulcal depth next to sulcal depths without p-tau immunoreactivity within the same tissue slide. In addition, prefrontal rostral cortex for case 3 displayed a focus of subpial p-tau in one sulcal depth with the CP13 and AT8 antibodies, but not the PHF1 antibody (Table 2). In cortical sulcal depths with p-tau immunoreactivity, GFAP immunoreactivity in serial tissue sections was likewise increased (Fig. 4a–f).

**Fig. 5:**
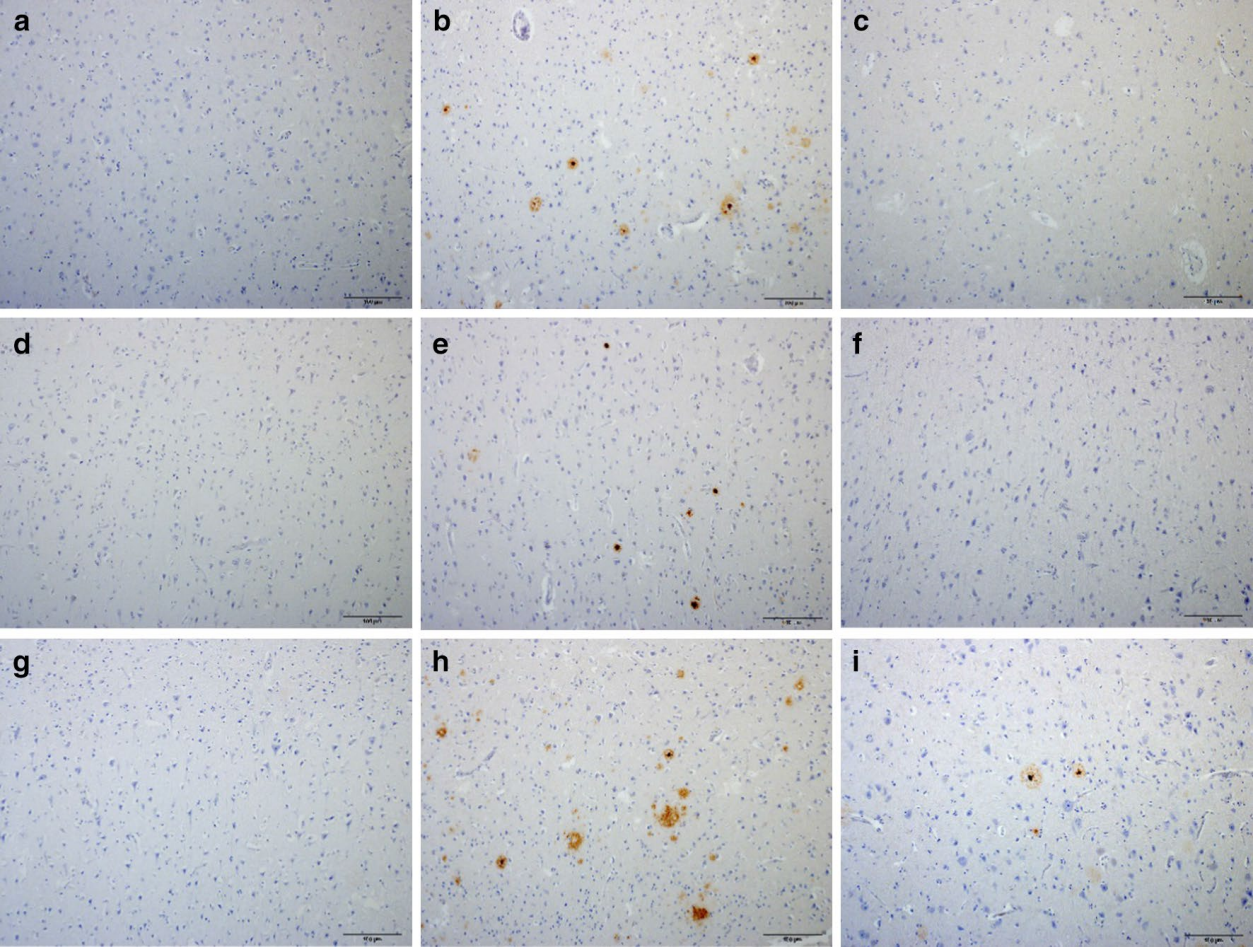
β-Amyloid pathology in cortex adjacent to leucotomy site. In leucotomized schizophrenia patients with *APOE* ε4 haplotype, β-amyloid plaques in cortex at leucotomy site (**b**, **e**, **h**), but minimal β-amyloid pathology in cortex rostral (**a**, **d**, **g**) or caudal (**c**, **f**, **i**) to the leucotomy site. Case 1 (**a**, **b**, **c**). Case 3 (**d**, **e**, **f**). Case 4 (**g**, **h**, **i**). β-amyloid immunohistochemistry (**a**–**i**). *Scale bars* **a**–**i**–100 μm

For cases 1, 3 and 4, β-amyloid immunoreactivity and modified Bielschowsky silver stains exhibited diffuse and cored plaques in gray matter at the leucotomy site, but no neuritic plaques or cerebral amyloid angiopathy (CAA) (Fig. 5b, e, h). Case 2 showed no evidence of β-amyloid pathology with either immunohistochemistry or Bielschowsky staining methods. Case 5, the oldest patient in the cohort at 87 years, displayed a small focus of cored and diffuse plaques with additional subpial immunoreactivity at one sulcal depth, which did not correspond to the p-tau immunoreactivity pattern and also differed from the β-amyloid plaques distributed throughout gray matter at the leucotomy site in cases 1, 3 and 4. *APOE* genotyping revealed ε4 haplotype for cases 1, 3 and 4 (ε3/4, ε2/4 and ε3/4, respectively), but genotypes ε3/3 for cases 2 and 5 (Table 3). Similar to the p-tau pathology patterns, cortical tissues rostral and caudal to the leucotomy site showed minimal β-amyloid pathology. The prefrontal rostral cortical samples from all five cases were negative for β-amyloid plaques (Fig. 5a, d, g). Case 1 showed minimal leptomeningeal CAA. For the frontal caudal cortical samples, cases 2 and 3 showed no evidence of β-amyloid pathology, whereas cases 4 and 5 exhibited few cored plaques in gray matter (Fig. 5c, f, i). Case 1 displayed one blood vessel with CAA in gray matter (Table 2). No β-amyloid immunoreactivity was detected in white matter.

**Table 3:** Apolipoprotein genotype of leucotomized and comparative non-leucotomized schizophrenia patients

Case number (leucotomy)	*APOE* genotype	Leucotomy site		Case number (no leucotomy)	*APOE* genotype
		β-Amyloid plaques	p-Tau		
1	ε3/4	+	+	6	ε3/3
2	ε3/3	−	+	7	ε3/4
3	ε2/4	+	+	8	ε3/4
4	ε3/4	+	+	9	ε3/3
5	ε3/3	−	+	10	ε3/4

For cases 1–5, positive (+) and negative (−) findings at the leucotomy site for both abnormally hyperphosphorylated tau in cortical neurons, astrocytes or cell processes, and β-amyloid plaques distributed throughout gray matter

*APOE* apolipoprotein E, *p*-*tau* abnormally hyperphosphorylated tau

**Fig. 6:**
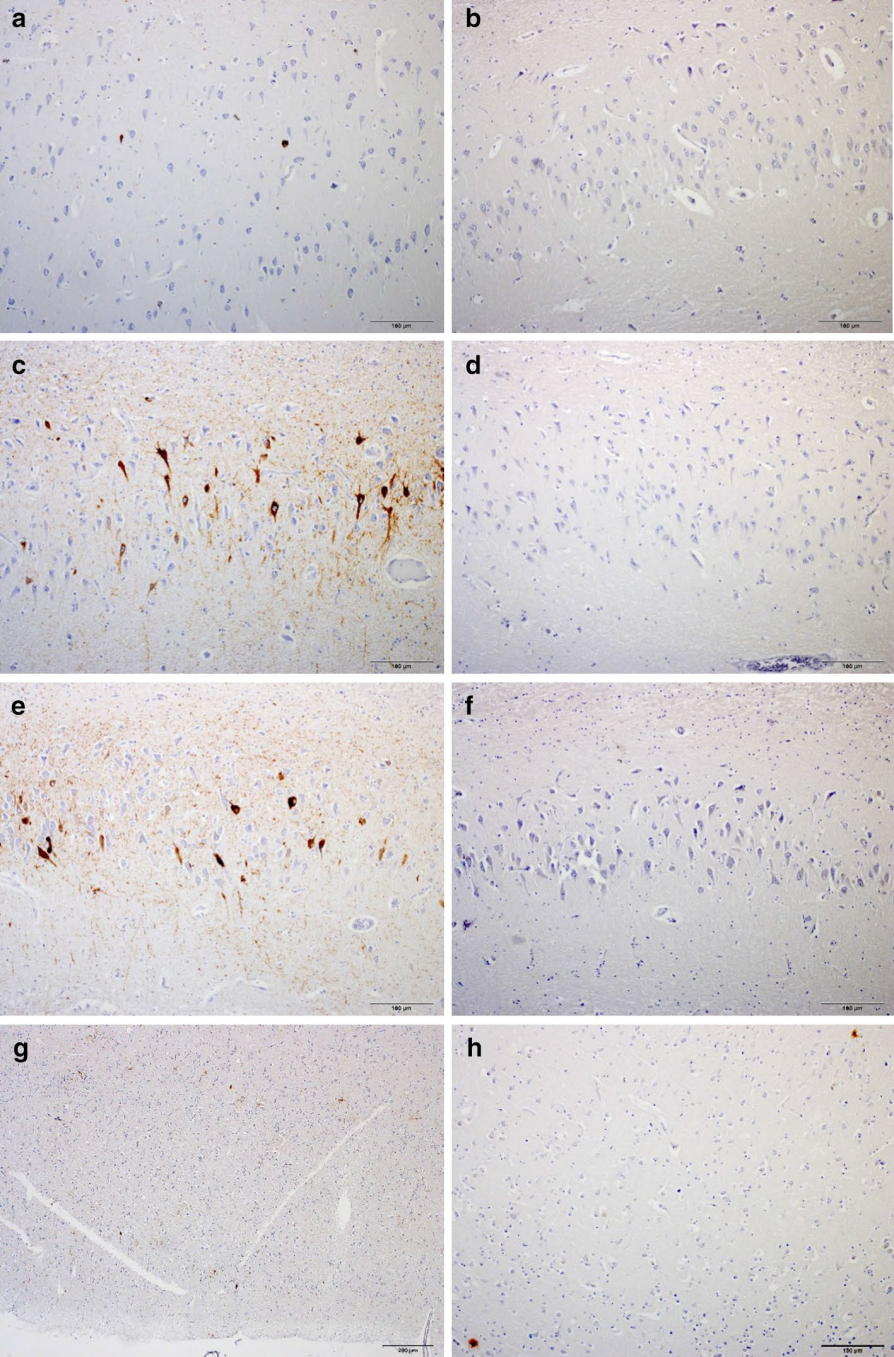
P-tau and β-amyloid pathology in hippocampus of leucotomized schizophrenia patients. Rare, isolated NFTs in CA1 of the two younger cases and moderate NFTs in CA1 of the three older cases (**a**, **c**, **e**), but no β-amyloid immunoreactivity (**b**, **d**, **f**). Several individual NFTs and β-amyloid plaques in the entorhinal cortex in the three older cases (**g**, **h**). Case 2 (**a**, **b**). Case 3 (**c**, **d**). Case 5 (**e**, **f**). AT8 (**a**, **c**, **e**, **g**) and β-amyloid (**b**, **d**, **f**, **h**) immunohistochemistry. *Scale bars* **a**–**f**, **h**; 100 μm, **e**; 200 μm

We also examined tissues sections from the hippocampus. In CTE, NFTs and other p-tau pathologies preferentially affect the CA2 and CA4 regions of the hippocampus, which differs from CA1 and subiculum involvement in normal aging or AD [26]. Cases 1 and 2 (ages 67 and 70 years, respectively) showed rare, isolated NFTs in CA1 and no β-amyloid plaques (Fig. 6a, b). Cases 3, 4 and 5 (ages 77, 87 and 89 years, respectively) revealed moderate NFTs in CA1, several individual NFTs in subiculum and entorhinal cortex, and diffuse and cored plaques in the entorhinal cortex (Fig. 6c–h). These p-tau and β-amyloid pathologies are consistent with aging, as opposed to CTE pathology.

### Schizophrenia patients without leucotomy

For comparison, we selected a cohort of five non-leucotomized schizophrenia patients who had lived in the same institution, matched in gender and approximate age (±4 years) at the time of death (mean age 77 years, range 67–86 years, 3 females and 2 males, Table 1). We studied brain specimens from identical cortical sites as the leucotomy patients. All tissue samples showed no significant pathology. GFAP and CD68 immunoreactivities were unremarkable. The cortical tissues revealed rare, scattered NFTs and β-amyloid plaques, but no p-tau encompassing blood vessels or favoring sulcal depths. Case 10 showed nominal CAA (Table 2). These five schizophrenia patients genotyped either as APOE ε3/3 or ε3/4 (Table 3).

## Discussion

In this study, we isolated the axonal injury component of human TBI by examining brain tissues from deceased patients with schizophrenia, who had undergone leucotomies at least 40 years before death. We specifically analyzed these tissues for chronic neurodegenerative sequelae. We provided evidence that massive chronic axonal damage in white matter of prefrontal cortex, as produced by leucotomy, results in accumulation of p-tau in neurons, astrocytes and cell processes, particularly around small blood vessels at depths of cortical sulci in irregular spatial patterns, in gray matter adjacent, but not rostral or caudal, to the axotomy site. This specific cortical p-tau distribution matches the recently established pathognomonic lesion of CTE, suggesting that axonal injury may serve as a pathophysiological substrate for this pathology over time, and possibly for both repetitive mild TBIs of contact sports and single severe or even moderate impact head injuries. Last, the brains of schizophrenia patients with leucotomy formed β-amyloid plaques in gray matter also adjacent to the axotomy site, but predominantly in those patients with the *APOE* ε4 haplotype. We did not observe comparable p-tau or β-amyloid accumulations in corresponding neuroanatomical prefrontal cortices as the axotomy sites in the non-leucotomized schizophrenia patients of matched age and gender, nor in corresponding rostral prefrontal or caudal frontal cortical sites.

CTE is a distinct neurodegenerative disorder linked to previous repetitive mild impact TBI and subconcussive events, classically described in retired boxers [4, 24]. The recent publication establishing initial diagnostic criteria for CTE points to p-tau accumulations in neurons, astrocytes and cell processes, favoring ensheathment of blood vessels at sulcal depths in irregular cortical patterns, as the pathognomonic lesion, thus distinguishing CTE from other neurodegenerative tauopathies and also aging-related tau astrogliopathy (ARTAG) [19, 26]. The underlying pathophysiology leading to this specific neuroanatomical distribution of p-tau aggregates, especially encompassing blood vessels, remains unanswered. There are two main hypotheses: that underlying axonal damage may be related to these pathognomonic lesions or that mechanical stress at sulcal depths during the traumatic event may render them susceptible to eventual p-tau deposition. Due to lack of extensive clinical history, we cannot comment upon head injury for an individual patient in this study. Nevertheless, because we observed p-tau immunoreactivity patterns similar to CTE only in the schizophrenia patients with leucotomy, which results in severe axonal injury without head impact, the data in this study support the hypothesis that selective p-tau accumulation at sulcal depths relates to underlying axonal damage.

Evidence exists to support the connection between traumatic axonal damage, which can persist for years, and accumulation of protein aggregates typically associated with neurodegenerative disease, with particular research focus on β-amyloid [14, 16, 43]. Pointedly, among those patients who had died or experienced temporal cortex surgical resection shortly after severe TBI, a subset showed β-amyloid plaques, including young people [10, 40, 41]. In diffuse axonal injury resultant of impact TBI, disruption of protein transport at sites of damage can lead to accumulation of proteins, including amyloid precursor protein (APP), in axonal varicosities and bulbs [16]. With axonal damage, proteins can abnormally congregate in the same space, either within the damaged axon or extracellular domain. As such, noxious molecular interactions potentially can occur, for example, β-secretase and presenilin-1 cleaving APP to generate β-amyloid that can then aggregate to form plaques [3, 16, 43, 49]. Likewise, indigenous tau proteins normally reside within axons, stabilizing neuronal microtubules to aid with axonal transport of essential components necessary for cell survival and function. With tauopathic neurodegenerative disease, tau proteins become hyperphosphorylated, disassociate from microtubules and aggregate in insoluble form. One postmortem study of human brain tissues, from persons who had died between 4 h and 5 weeks after TBI, showed PHF1 immunopositivity in axonal varicosities and bulbs, neuronal cell bodies and reactive astrocytes within the cerebral cortex in two of 18 cases [49]. In the TBI study of surgically resected temporal cortices, tissue samples in 12 of 18 cases displayed dystrophic axons in white matter with positive PHF1 immunoreactivity; three cases showed PHF1 immunopositive glia, and four cases revealed PHF1 immunopositive neurons [10]. For the schizophrenia patients who had survived at least 40 years after leucotomy in this study, we similarly noted NFTs, astrocytic tangles and p-tau immunoreactive cell processes in neuroanatomical areas near the axotomy site, with three p-tau antibodies including PHF1. In addition, we observed β-amyloid plaques scattered in the gray matter within proximity of the massive axonal damage in a subset of the leucotomized cases (*APOE* ε4 haplotype). Given these data and literature, we surmise that damaged axons may inadvertently release proteins at the lesion site into the extracellular brain parenchyma, perhaps over long periods of time, thereby promoting aberrant molecular reactions and deleterious aggregation of particular proteins.

One possible explanation for the p-tau accumulation pattern in CTE, and the leucotomized patients in this study, lies in interstitial solute clearance in the human brain through local diffusion and fluid bulk flow, a process first proposed about 100 years ago with recent scientific breakthroughs [12]. The human brain lacks characteristic lymphatic vessels, utilized by the rest of the body, to provide protein and fluid homeostasis. In contemporary experiments involving rodents, data show that cerebrospinal fluid (CSF) in the subarachnoid space influxes through periarterial spaces and exits through the aquaporin 4 channels of astrocytic endfeet, mixes with interstitial fluid to flow by convection through the interstitial space clearing macromolecules and finally passes through aquaporin 4 channels of astrocytic endfeet into perivenous spaces—termed the glymphatic pathway for the dependence upon glial cells to function as the surrogate lymphatic system in the brain [30]. The perivenous fluid effluxes to contiguous CSF that filtrates through arachnoid granulations to dural sinuses for venous drainage. Two recent papers provide evidence that CSF, with its soluble and cellular contents, flows into newly discovered lymphatic vessels in meninges lining dural sinuses that drain into cervical lymph nodes [1, 23], in addition to lymphatic vessels of nasal mucosa, cranial nerve sheathes and particular arteries and veins exiting the cranium [1]. In another paper employing mouse models, the authors showed impaired function of the glymphatic system after TBI, with prominent astrogliosis, loss of perivascular aquaporin 4 polarization and axonal damage and then specifically identified diminished interstitial tau clearance with accompanying hyperphosphorylated tau increase [11]. One common observation reported in CTE, and also in the leucotomy patients of this study, is p-tau accumulation encompassing blood vessels and in perivascular astrocytes, which is consistent with clearance data in rodents. Considered a biomarker for axonal injury, total tau levels increase in CSF and blood serum after TBI in humans, including contact sports athletes shortly after mild head injuries [31, 42, 50]. Nevertheless, since most experiments involve rodents, with lissencephalic brain structures, the specific mechanisms of clearance within the human brain, particularly its relation to sulcal depths, remain poorly understood. We suspect that the sulcal depth serves as an exodus for tau clearance after axonal damage in humans. If true, then axonal injury, which occurs with TBI ranging from mild to severe, may account for the pathognomonic p-tau signature pattern of CTE [26]. Finally, brain tissues from long-term survivors of single moderate to severe TBI, with presumable axonal injuries that can persist for years, also display NFTs and p-tau immunoreactive astrocytes in cortical sulcal depths [14, 17]. Taken together, these data and literature not only bolster the link between head trauma and CTE, but also attenuate the notion of the pathognomonic CTE lesion being limited to multiple mild TBIs and/or subconcussive events.

CTE is classified as a tauopathy, yet a subset of reported cases displays diffuse or neuritic β-amyloid plaques in cortical gray matter, which instigated the debate decades ago concerning the pathophysiologic connections among head injury, neuropathological neurodegenerative indicators such as p-tau and β-amyloid protein accumulations and augmented development of neurodegenerative diseases. In 1973, after extensive examination of brain tissues from 15 deceased boxers, Corsellis, et al. reported only one case with neuritic β-amyloid plaques, using classic silver staining techniques [4]. Interestingly, Roberts, et al. analyzed brain tissues from 14 of these same cases plus six others, but additionally employed immunohistochemistry experiments for β-amyloid detection, to discover numerous diffuse plaques in most cases [39]. Moreover, several papers provide data suggesting an association between the *APOE* ε4 genotype and predilection for β-amyloid deposition in the brains of patients dying shortly after head injury [9, 32] and also in CTE patients especially with advancing years [44]. In this present study, all three leucotomized patients with the *APOE* ε4 haplotype displayed diffuse and cored β-amyloid plaques scattered throughout the gray matter adjacent to the lesion site, in contrast to the leucotomized patients with the *APOE* ε3/3 genotype. The oldest leucotomized patient, who had died at 87 years with an *APOE* ε3/3 genotype, showed a focus of cored β-amyloid plaques at one sulcal depth, which complements a recent study reporting predilection of β-amyloid aggregates at cortical sulcal depths in CTE patients [44]. All five schizophrenia patients without leucotomies showed minimal or no β-amyloid immunoreactivity in cortical gray matter, including three patients with *APOE* ε4 haplotype. The molecular interaction between ApoE protein and β-amyloid warrants further clarification; however, ApoE can bind β-amyloid to enhance its clearance through multiple mechanisms including proteolysis, with ApoE E_4_ as the least effective isoform [22]. In mouse models, experimental data demonstrated that murine brains clear soluble β-amyloid along glymphatic paravenous drainage pathways as well and that deletion of the *Aqp4* gene attenuates this process [13]. In addition to the glymphatic system for clearance of β-amyloid, efflux transporters, such as the low-density lipoprotein receptor-related protein-1 (LRP1), facilitate transendothelial transport across the blood–brain barrier [51, 52]. Human brain studies revealed cerebrovascular β-amyloid deposits in three former boxers and increased frequency of CAA in *APOE* ε4 patients with severe acute TBI [21, 48]. In this study, we observed minimal CAA or perivascular β-amyloid aggregation in all ten schizophrenia patients. Rather, brain tissues of the *APOE* ε4 haplotype patients with leucotomy showed β-amyloid plaques throughout gray matter overlying the axotomy site.

This study has several limitations in design and interpretation. Foremost, we lacked detailed antemortem clinical data, including head injury history, which curtailed the extent of neuropathological analysis. Additionally, we assessed brain tissues from two small cohorts: five schizophrenia patients with leucotomy and for comparison, five schizophrenia patients without leucotomy who had also lived at the Pilgrim Psychiatric Center during approximately the same time period. Most likely aging in the schizophrenic brain diverges from persons without this condition, as evidenced by schizophrenia patients in advanced years more frequently suffering from severe cognitive impairments than the general population [37, 38]. A neuropathological study of 100 consecutive autopsy patients (age range, 52–101 years; mean, 77 years), however, shows equivalent amounts of β-amyloid plaques and NFTs in neocortex and hippocampus of patients with schizophrenia, as compared to 47 patients with other psychiatric disorders and 50 patients without psychiatric or dementing diagnoses, when matched by age [37]. The study authors concluded that the percentage of elderly schizophrenia patients with AD, as determined by neuropathological criteria, basically equates to the general elderly population with corresponding ages. Since schizophrenia involves neurodevelopmental abnormalities, the possibility exists that clearance of p-tau, β-amyloid and other neurodegenerative proteins may differ from persons without schizophrenia. Due to the small number of patients in each cohort, uncertainty of tissue integrity equivalence from archived brain specimens and tendency for focality and irregularity patterns of especially the p-tau immunoreactivity, we did not numerically quantify pathological results. Also, because we obtained specimens previously dissected for other studies, we were unable to analyze hemibrain sections similar to contemporary CTE studies to attain more thorough neuroanatomical distribution assessment of p-tau aggregates in sulcal depths. As such, we could not determine potential cortical spatial patterns for sulcal tauopathy, which currently are described as irregular in CTE. Furthermore, given these limiting circumstances, we may have serendipitously missed sulcal tauopathy in the sampling of brain tissues from case 1. Other possibilities for lack of sulcal p-tau pathology in case 1 include that this individual responded differently than the other four patients after the leucotomy procedure, or that since this patient died at the youngest age (67 years), with more prolonged life this patient may have accumulated p-tau in sulcal depths similar to the other older leucotomized patients in the cohort.

In summary, we studied a human model of severe axonal injury, analyzing brain tissues specifically for chronic neurodegenerative changes. We found p-tau pathology in patterns resembling CTE in gray matter adjacent to the leucotomy site. We also observed a subset of cases with β-amyloid plaques, similarly reported in CTE, scattered in gray matter at the leucotomy site, but in patients with *APOE* ε4 haplotype, which is associated with worse clinical outcomes for TBI patients [6, 25, 47], including boxers and American football players [18, 20]. These data suggest that axonal injury, common to all severities of TBI, contributes to the distinct pathology of CTE over time.

## References

[1] Aspelund A, Antila S, Proulx ST, Karlsen TV, Karaman S, Detmar M, Wiig H, Alitalo K (2015). A dural lymphatic vascular system that drains brain interstitial fluid and macromolecules. J Exp Med.

[2] Calero O, Hortiguela R, Bullido MJ, Calero M (2009). Apolipoprotein E genotyping method by real time PCR, a fast and cost-effective alternative to the TaqMan and FRET assays. J Neurosci Methods.

[3] Chen XH, Johnson VE, Uryu K, Trojanowski JQ, Smith DH (2009). A lack of amyloid beta plaques despite persistent accumulation of amyloid beta in axons of long-term survivors of traumatic brain injury. Brain Pathol.

[4] Corsellis JA, Bruton CJ, Freeman-Browne D (1973). The aftermath of boxing. Psychol Med.

[5] Freeman W, Watts JW (1950). Psychosurgery: in the treatment of mental disorders and intractable pain.

[6] Friedman G, Froom P, Sazbon L, Grinblatt I, Shochina M, Tsenter J, Babaey S, Yehuda B, Groswasser Z (1999). Apolipoprotein E-epsilon4 genotype predicts a poor outcome in survivors of traumatic brain injury. Neurology.

[7] Guo Z, Cupples LA, Kurz A, Auerbach SH, Volicer L, Chui H, Green RC, Sadovnick AD, Duara R, DeCarli C (2000). Head injury and the risk of AD in the MIRAGE study. Neurology.

[8] Hirano A, Zimmerman HM (1962). Silver impregnation of nerve cells and fibers in celloidin sections. A simple impregnation technique. Arch Neurol.

[9] Horsburgh K, Cole GM, Yang F, Savage MJ, Greenberg BD, Gentleman SM, Graham DI, Nicoll JA (2000). beta-amyloid (Abeta)42(43), abeta42, abeta40 and apoE immunostaining of plaques in fatal head injury. Neuropathol Appl Neurobiol.

[10] Ikonomovic MD, Uryu K, Abrahamson EE, Ciallella JR, Trojanowski JQ, Lee VM, Clark RS, Marion DW, Wisniewski SR, DeKosky ST (2004). Alzheimer’s pathology in human temporal cortex surgically excised after severe brain injury. Exp Neurol.

[11] Iliff JJ, Chen MJ, Plog BA, Zeppenfeld DM, Soltero M, Yang L, Singh I, Deane R, Nedergaard M (2014). Impairment of glymphatic pathway function promotes tau pathology after traumatic brain injury. J Neurosci.

[12] Iliff JJ, Goldman SA, Nedergaard M (2015). Implications of the discovery of brain lymphatic pathways. Lancet Neurol.

[13] Iliff JJ, Wang M, Liao Y, Plogg BA, Peng W, Gundersen GA, Benveniste H, Vates GE, Deane R, Goldman SA (2012). A paravascular pathway facilitates CSF flow through the brain parenchyma and the clearance of interstitial solutes, including amyloid beta. Sci Transl Med.

[14] Johnson VE, Stewart JE, Begbie FD, Trojanowski JQ, Smith DH, Stewart W (2013). Inflammation and white matter degeneration persist for years after a single traumatic brain injury. Brain.

[15] Johnson VE, Stewart W, Smith DH (2013). Axonal pathology in traumatic brain injury. Exp Neurol.

[16] Johnson VE, Stewart W, Smith DH (2010). Traumatic brain injury and amyloid-beta pathology: a link to Alzheimer’s disease?. Nat Rev Neurosci.

[17] Johnson VE, Stewart W, Smith DH (2012). Widespread tau and amyloid-beta pathology many years after a single traumatic brain injury in humans. Brain Pathol.

[18] Jordan BD, Relkin NR, Ravdin LD, Jacobs AR, Bennett A, Gandy S (1997). Apolipoprotein E epsilon4 associated with chronic traumatic brain injury in boxing. JAMA.

[19] Kovacs GG, Ferrer I, Grinberg LT, Alafuzoff I, Attems J, Budka H, Cairns NJ, Crary JF, Duyckaerts C, Ghetti B (2016). Aging-related tau astrogliopathy (ARTAG): harmonized evaluation strategy. Acta Neuropathol.

[20] Kutner KC, Erlanger DM, Tsai J, Jordan B, Relkin NR (2000). Lower cognitive performance of older football players possessing apolipoprotein E epsilon4. Neurosurg.

[21] Leclercq PD, Murray LS, Smith C, Graham DI, Nicoll JA, Gentleman SM (2005). Cerebral amyloid angiopathy in traumatic brain injury: association with apolipoprotein E genotype. J Neurol Neurosurg Psychiatry.

[22] Liu CC, Kanekiyo T, Xu H, Bu G (2013). Apolipoprotein E and Alzheimer disease: risk, mechanisms and therapy. Nat Rev Neurol.

[23] Louveau A, Smirnov I, Keyes TJ, Eccles JD, Rouhani SJ, Peske JD, Derecki NC, Castle D, Mandell JW, Lee KS (2015). Structural and functional features of central nervous system lymphatic vessels. Nature.

[24] Martland HS (1928). Punch drunk. JAMA.

[25] Mayeux R, Ottman R, Maestre G, Ngai C, Tang MX, Ginsberg H, Chun M, Tycko B, Shelanski M (1995). Synergistic effects of traumatic head injury and apolipoprotein-epsilon 4 in patients with Alzheimer’s disease. Neurol.

[26] McKee AC, Cairns NJ, Dickson DW, Folkerth RD, Keene CD, Litvan I, Perl DP, Stein TD, Vonsattel JP, Stewart W (2016). The first NINDS/NIBIB consensus meeting to define neuropathological criteria for the diagnosis of chronic traumatic encephalopathy. Acta Neuropathol.

[27] McKee AC, Cantu RC, Nowinski CJ, Hedley-Whyte ET, Gavett BE, Budson AE, Santini VE, Lee HS, Kubilus CA, Stern RA (2009). Chronic traumatic encephalopathy in athletes: progressive tauopathy after repetitive head injury. J Neuropathol Exp Neurol.

[28] McKee AC, Stein TD, Nowinski CJ, Stern RA, Daneshvar DH, Alvarez VE, Lee HS, Hall G, Wojtowicz SM, Baugh CM (2013). The spectrum of disease in chronic traumatic encephalopathy. Brain.

[29] Mortimer JA, van Duijn CM, Chandra V, Fratiglioni L, Graves AB, Heyman A, Jorm AF, Kokmen E, Kondo K, Rocca WA (1991). Head trauma as a risk factor for Alzheimer’s disease: a collaborative re-analysis of case-control studies. EURODEM Risk Factors Research Group. Int J Epidemiol.

[30] Nedergaard M (2013). Neuroscience. Garbage truck of the brain. Science.

[31] Neselius S, Brisby H, Theodorsson A, Blennow K, Zetterberg H, Marcusson J (2012). CSF-biomarkers in Olympic boxing: diagnosis and effects of repetitive head trauma. PLoS One.

[32] Nicoll JA, Roberts GW, Graham DI (1995). Apolipoprotein E epsilon 4 allele is associated with deposition of amyloid beta-protein following head injury. Nat Med.

[33] Omalu B, Bailes J, Hamilton RL, Kamboh MI, Hammers J, Case M, Fitzsimmons R (2011). Emerging histomorphologic phenotypes of chronic traumatic encephalopathy in American athletes. Neurosurg.

[34] Oppenheimer DR (1968). Microscopic lesions in the brain following head injury. J Neurol Neurosurg Psychiatry.

[35] Plassman BL, Havlik RJ, Steffens DC, Helms MJ, Newman TN, Drosdick D, Phillips C, Gau BA, Welsh-Bohmer KA, Burke JR (2000). Documented head injury in early adulthood and risk of Alzheimer’s disease and other dementias. Neurol.

[36] Injury Prevention & Control: Traumatic Brain Injury & Concussion. https://www.cdc.gov/traumaticbraininjury/get_the_facts.html. Accessed 1 Oct 2016

[37] Purohit DP, Perl DP, Haroutunian V, Powchik P, Davidson M, Davis KL (1998). Alzheimer disease and related neurodegenerative diseases in elderly patients with schizophrenia: a postmortem neuropathologic study of 100 cases. Arch Gen Psychiatry.

[38] Rapp MA, Schnaider-Beeri M, Purohit DP, Reichenberg A, McGurk SR, Haroutunian V, Harvey PD (2010). Cortical neuritic plaques and hippocampal neurofibrillary tangles are related to dementia severity in elderly schizophrenia patients. Schizophr Res.

[39] Roberts GW, Allsop D, Bruton C (1990). The occult aftermath of boxing. J Neurol Neurosurg Psychiatry.

[40] Roberts GW, Gentleman SM, Lynch A, Graham DI (1991). beta A4 amyloid protein deposition in brain after head trauma. Lancet.

[41] Roberts GW, Gentleman SM, Lynch A, Murray L, Landon M, Graham DI (1994). Beta amyloid protein deposition in the brain after severe head injury: implications for the pathogenesis of Alzheimer’s disease. J Neurol Neurosurg Psychiatry.

[42] Shahim P, Tegner Y, Wilson DH, Randall J, Skillback T, Pazooki D, Kallberg B, Blennow K, Zetterberg H (2014). Blood biomarkers for brain injury in concussed professional ice hockey players. JAMA Neurol.

[43] Smith DH, Johnson VE, Stewart W (2013). Chronic neuropathologies of single and repetitive TBI: substrates of dementia?. Nat Rev Neurol.

[44] Stein TD, Montenigro PH, Alvarez VE, Xia W, Crary JF, Tripodis Y, Daneshvar DH, Mez J, Solomon T, Meng G (2015). Beta-amyloid deposition in chronic traumatic encephalopathy. Acta Neuropathol.

[45] Stern RA, Daneshvar DH, Baugh CM, Seichepine DR, Montenigro PH, Riley DO, Fritts NG, Stamm JM, Robbins CA, McHale L (2013). Clinical presentation of chronic traumatic encephalopathy. Neurol.

[46] Strich SJ (1956). Diffuse degeneration of the cerebral white matter in severe dementia following head injury. J Neurol Neurosurg Psychiatry.

[47] Teasdale GM, Nicoll JA, Murray G, Fiddes M (1997). Association of apolipoprotein E polymorphism with outcome after head injury. Lancet.

[48] Tokuda T, Ikeda S, Yanagisawa N, Ihara Y, Glenner GG (1991). Re-examination of ex-boxers’ brains using immunohistochemistry with antibodies to amyloid beta-protein and tau protein. Acta Neuropathol.

[49] Uryu K, Chen XH, Martinez D, Browne KD, Johnson VE, Graham DI, Lee VM, Trojanowski JQ, Smith DH (2007). Multiple proteins implicated in neurodegenerative diseases accumulate in axons after brain trauma in humans. Exp Neurol.

[50] Zetterberg H, Hietala MA, Jonsson M, Andreasen N, Styrud E, Karlsson I, Edman A, Popa C, Rasulzada A, Wahlund LO (2006). Neurochemical aftermath of amateur boxing. Arch Neurol.

[51] Zhao Z, Sagare AP, Ma Q, Halliday MR, Kong P, Kisler K, Winkler EA, Ramanathan A, Kanekiyo T, Bu G (2015). Central role for PICALM in amyloid-beta blood-brain barrier transcytosis and clearance. Nat Neurosci.

[52] Zlokovic BV, Deane R, Sagare AP, Bell RD, Winkler EA (2010). Low-density lipoprotein receptor-related protein-1: a serial clearance homeostatic mechanism controlling Alzheimer’s amyloid beta-peptide elimination from the brain. J Neurochem.

